# The *hrp* genes of *Pseudomonas cichorii* are essential for pathogenicity on eggplant but not on lettuce

**DOI:** 10.1099/mic.0.2008/021097-0

**Published:** 2008-10

**Authors:** Hiroshi Hojo, Makoto Koyanagi, Masayuki Tanaka, Shigeru Kajihara, Kouhei Ohnishi, Akinori Kiba, Yasufumi Hikichi

**Affiliations:** 1 Laboratory of Plant Pathology and Biotechnology, Kochi University, 200 Monobe, Nankoku, Kochi 783-8502, Japan; 2 Institute of Molecular Genetics, Kochi University, 200 Monobe, Nankoku, Kochi 783-8502, Japan

## Abstract

*Pseudomonas cichorii* causes necrotic lesions in eggplant and rot in lettuce. Through transposon insertion into *P. cichorii* strain SPC9018 we produced two mutants, 4-57 and 2-99, that lost virulence on eggplant but not lettuce. Analyses showed that a transposon was inserted into the *hrpG* gene in 4-57 and the *hrcT* gene in 2-99. Nucleotide sequences of the *hrp* genes of SPC9018 are homologous to those of *Pseudomonas viridiflava* BS group strains. The pathogenicity of 4-57 on eggplant was restored by transformation with an *hrpF* operon, originating from either SPC9018 or the BS group member *P. viridiflava* strain 9504 (Pv9504). These data suggested the involvement of *hrp* genes in the pathogenicity of SPC9018 on eggplant, and functional conservation of *hrpF* operons between SPC9018 and Pv9504. Both the *hrpS* mutant and the *hrpL* mutant were unable to cause necrotic lesions on eggplant leaves but retained their pathogenicity against lettuce. These results suggest that the pathogenicity of *P. cichorii* is *hrp*-dependent in eggplant, but not in lettuce.

## INTRODUCTION

*Pseudomonas cichorii* causes necrotic leaf spot on eggplants and rot on lettuce. Bacterial rot caused by the bacteria is called ‘varnish spot’ in California or ‘tar’ in Japan. The rot is characterized by shiny, dark-brown, firm necrotic spots on leaves underneath the second or the third outermost head leaves. Microscopic observations using *lux*-marked *P. cichorii* and an immunofluorescent antibody against the bacteria have shown that the bacteria first invade lettuce head leaves through stomata and then colonize intercellular spaces (Hikichi *et al.*, 1996a, 1998). After proliferation in intercellular spaces of the mesophyll, the bacteria spread throughout the whole leaf via the vascular bundle (Hikichi *et al.*, 1996b). The symptoms appear and develop as the bacteria multiply and spread. Our previous studies have shown that *de novo* protein synthesis in lettuce leaves is required for the development of disease symptoms (Hikichi *et al.*, 1998; Kiba *et al.*, 2006a). Moreover, the development of disease symptoms is closely associated with programmed cell death (PCD), following heterochromatin aggregation and laddering of genome DNA in the *P. cichorii*-infected lettuce cells (Kiba *et al.*, 2006a). *P. cichorii* causes necrotic spot on eggplant, sweet pepper, celery and okra, distinct from the disease symptoms on lettuce. Kiba *et al.* (2006b) have shown that development of necrotic lesions following PCD on leaves of eggplants inoculated with *P. cichorii* is commonly associated with *de novo* protein synthesis, intracellular reactive oxygen species and caspase III-like protease activity.

In several Gram-negative phytopathogenic bacteria, the *hrp* genes are essential determinants for disease development on compatible hosts and for elicitation of the hypersensitive response (HR) on resistant plants (Alfano & Collmer, 1997). The *hrp* genes encode proteins in the type III secretion system (TTSS), which is believed to transport virulence proteins directly into the host cells. These proteins subsequently cause leakage of plant nutrients into the extracellular spaces of infected tissues and suppress host defences. Nine of the *hrp* genes have been renamed *hrc* (HR and conserved) to indicate that they encode conserved components that are also present in the type III secretion machinery of the animal pathogens *Yersinia*, *Shigella* and *Salmonella* (Bogdanove *et al.*, 1996). Recently Araki *et al.* (2006) have reported that *hrp* genes exist in the genomic DNA of *P. cichorii* strain 83-1, but the roles of these genes in the pathogenicity of *P. cichorii* remain unclear.

In this study, we isolated two mutants that lost virulence on eggplants after transposon mutagenesis of *P. cichorii* strain SPC9018 (SPC9018). However, the mutants retained their virulence on lettuce. Molecular analysis revealed that the transposons were inserted in the *hrpG* and *hrcT* genes. Pathogenicity analysis using *hrp* mutants showed that the *hrp* genes of *P. cichorii* are essential for its ability to cause necrotic lesion symptoms on eggplant, but not to cause rot symptoms on lettuce.

## METHODS

### Bacterial strains, culture conditions and plasmids.

Bacterial strains and plasmids are listed in Table 1. *P. cichorii* strains were routinely grown in PS medium (Wakimoto *et al.*, 1968) at 30 °C. *Escherichia coli* strains were grown in LM medium (Hanahan, 1983) at 37 °C. Ampicillin (50 μg ml^−1^), kanamycin (50 μg ml^−1^) and tetracycline (30 μg ml^−1^) were used in selective media. Populations of SPC9018 and mutants *in planta* were assayed in three independent experiments using PCSM plates (Uematsu *et al.*, 1982) and PCSM plates containing kanamycin.

### General DNA manipulations.

Isolation of genomic DNA, plasmid DNA manipulations, PCR and Southern blot analyses were performed using standard techniques (Sambrook *et al.*, 1989). *P. cichorii* was transformed by electroporation as described by Allen *et al.* (1991). Double-stranded DNA sequencing templates were prepared with GenElute Plasmid Miniprep kits (Sigma Chemical). Sequences were determined using an Automated DNA Sequencer model 373 (Applied Biosystems). DNA sequence data were analysed using the dnasis-Mac software (Hitachi Software Engineering). Enzymes including restriction endonucleases (Takara) were used according to the manufacturer's instructions.

### Transposon mutagenesis.

To create *P. cichorii* mutants with transposon insertions, 0.5 μg placZ2 (de Lorenzo *et al.*, 1990) containing mini-Tn*5*lacZ2 was used for electroporation of SPC9018 competent cells. The EZ : TN transposome-mediated insertion system (Epicentre) was also used (Tsujimoto *et al.*, 2008). The transposon EZ : : TN <KAN-2> (0.1 μg) was mixed with an equal volume of 100 % glycerol and 2 μl EZ : TN transposase (1 unit μl^−1^), and incubated at 37 °C for 10 min. Aliquots (1 μl) were used for electroporation of *P. cichorii*. Transposition clones were selected by plating on PS medium containing kanamycin.

### Transposon-inserted site in genomic DNA of mutants.

To determine whether a single transposon insertion had occurred in the genome of mutants 2-99 and 4-57 through the insertion by EZ : : Tn <KAN-2> and mini-Tn*5*lacZ2, respectively, their genomic DNAs were isolated using the AquaPure Genomic DNA Isolation kit (Bio-Rad). DNA from 2-99 was digested with *Eco*RI, 4-57 DNA was digested with *Xho*I, and the DNA fragments were then separated by agarose gel electrophoresis and hybridized with Km^R^ from EZ : : Tn <KAN-2> (2-99) and Km^R^ from pUCK191 (4-57) (Tsuge *et al.*, 2001).

The *Kpn*I fragments from genomic DNA of the mutants were ligated into the plasmid vector pUC118 (Takara), and the resulting plasmids were transformed into *E. coli* DH5*α* (Takara). Kanamycin-resistant transformants were isolated. Each harboured plasmid p2-99 or p4-57, which contained 13.9 or 5.6 kb DNA fragments from genomic DNA of 2-99 and 4-57, respectively. The inserts carried on p2-99 and p4-57 were also sequenced.

### Determination of the nucleotide sequence of the *hrp* cluster.

To create a genomic library of SPC9018, genomic DNA from SPC9018 was isolated and partially digested by *Sau*3AI. DNA fragments (10–20 kb) were collected by sucrose density-gradient centrifugation (Kanda *et al.*, 2003). The DNA fragments were ligated into the *Bam*HI site of pBluescript II KS+ (Stratagene), and transformed into *E. coli* DH5*α*, to create a genomic library of SPC9018.

A 703 bp *Sph*I- and *Xho*I-digested fragment of p2-99, and a 1.1 kb *Hin*dIII- and *Sma*I-digested fragment of p4-57 were ligated into pUC118 to create plasmids pP2-99 and pP4-57, respectively. The plasmids were used as templates in PCRs. Southern blot hybridization was performed using DIG-labelled DNA probes (Roche Molecular Biochemicals) to probe the *hrp* cluster from the SPC9018 genomic library. The DIG-labelled probes were PCR-amplified with M4 (5′-GTTTTCCCAGTCACGAC-3′) and RV (5′-CAGGAAACAGCTATGAC-3′) according to the manufacturer's protocol. The resultant positive transformant hrpG-posi harboured the plasmid pHOJO containing a 30 kb insert. The other positive transformant, hrcT-posi, harboured pKAJI containing a 24.3 kb insert. These were used to sequence the *hrp* cluster.

### Complementation of 4-57 with *hrpF* operons.

A 3.6 kb fragment was PCR-amplified from the genomic DNA of SPC9018 with the following primers: 5′-GCTCTAGAGGGTCAACTGGGCTGGACGTTG-3′ (named Xba-FW-hrpFoperon) with an added *Xba*I site (underlined) and 5′-GCTCTAGATGCGCGTCGCGTTGAGAGTTCG-3′ (named Xba-RV-hrpFoperon) with an added *Xba*I site (underlined). PCR amplification was performed with 1 cycle of 94 °C for 2 min, 5 cycles of 94 °C for 1 min, 62 °C for 1 min, and 72 °C for 3 min, 20 cycles of 94 °C for 1 min, 65 °C for 1 min, and 72 °C for 3 min. The *Xba*I-digested fragment was ligated into the *Xba*I site of pLAFR3 (Staskawicz *et al.*, 1987), and phrpFoperon was created. A 4.1 kb fragment was PCR-amplified from the genomic DNA of *Pseudomonas viridiflava* strain 9504 (Pv9504), which belongs to the BS group, with the following primers: 5′-GCTCTAGACTCATGTTGACCCGTCGCAGTC-3′ (named Xba-PV-FW-hrpFoperon) with an added *Xba*I site (underlined) and 5′-GCTCTAGAGCATGTCGCGTTGGGAGTTCGC-3′ (named Xba-PV-RV-hrpFoperon) with an added *Xba*I site (underlined), based on the nucleotide sequences of *P. viridiflava* strains ME3.1b, RMX23.1a and RMX3.1b (Araki *et al.*, 2006). PCR amplification was performed with 1 cycle of 94 °C for 2 min, 5 cycles of 94 °C for 1 min, 60 °C for 1 min, and 72 °C for 3 min, 20 cycles of 94 °C for 1 min, 65 °C for 1 min, and 72 °C for 3 min. The *Xba*I-digested fragment was ligated into the *Xba*I site of pLAFR3, and phrpFoperonPV was created. phrpFoperon and phrpFoperonPV were transformed into 4-57 competent cells, and the tetracycline-resistant transformants 4-57F and 4-57FPV were created, respectively.

### Creation of *hrpL* and *hrpS* mutants.

An 870 bp fragment (named 3-4) was PCR-amplified from the genomic DNA of SPC9018 with the following primers: 5′-CAGCCCTGCAGAACGCTAAC-3′ (named 4-Pst) and 5′-CGAGCTCTGAACAGTTTTGTCCC-3′ (named 3-Sac) with an added *Sac*I site (underlined). PCR amplification was performed with 1 cycle of 94 °C for 2 min, 5 cycles of 94 °C for 1 min, 50 °C for 1 min, and 72 °C for 1 min, 20 cycles of 94 °C for 1 min, 55 °C for 1 min, and 72 °C for 1 min. The *Sac*I- and *Pst*I-digested 3-4 fragment was ligated into the *Sac*I and *Pst*I sites of pHSG398 (Takara), and pL3-4 was created. A 464 bp fragment (named 1-2) was PCR-amplified from the genomic DNA of SPC9018 with the following primers: 5′-GGAATTCGGGGCGTTCCACGCTTTC-3′ (named 1-RI) with an added *Eco*RI site (underlined) and 5′-GGGAGCTCGGTCACTGCATGCCTTTGACTTC-3′ (named 2-Sac) with an added *Sac*I site (underlined). PCR amplification was performed with 1 cycle of 94 °C for 2 min, 5 cycles of 94 °C for 1 min, 50 °C for 1 min, and 72 °C for 30 s, 20 cycles of 94 °C for 1 min, 55 °C for 1 min, and 72 °C for 30 s. The *Eco*RI- and *Sac*I-digested 1-2 fragment was ligated into the *Eco*RI and *Sac*I sites of pL3-4, and phrpL was created. A 1.4 kb *Kpn*I-digested fragment of pUCK191 containing Km^R^ was blunt-ended by T4 DNA polymerase and ligated into the blunt-ended phrpL *Sac*I site to create p398-LKm. A 2.6 kb *Pst*I- and *Bam*HI-digested DNA fragment containing *sacB* from pUCD800 (Gay *et al.*, 1985) was blunt-ended by T4 DNA polymerase and ligated into the blunt-ended p398-LKm *Eco*RI site to create p398-LKmsac. This plasmid was electroporated into SPC9018 cells and the resulting kanamycin- and sucrose-resistant recombinant, SPC9018-L, was selected. Southern blot analysis was performed to verify correct insertion of the 2.7 kb fragment containing Km^R^ into the *hrpL* locus in the genetic backgrounds isolated (data not shown), and this showed that SPC9018-L was an *hrpL*-deficient mutant of SPC9018.

A 4.7 kb *Eco*RI- and *Sma*I-digested fragment of pHOJO was ligated into the *Eco*RI and *Sma*I sites of pHSG398, and p398-S was created. A 1.4 kb *Kpn*I-digested fragment of pUCK191 containing Km^R^ was blunt-ended by T4 DNA polymerase and inserted into the the p398-S *Eco*RV site to create p398-SKm. A 2.6 kb *Bam*HI- and *Pst*I-digested DNA fragment containing *sacB* from pUCD800 was blunt-ended by T4 DNA polymerase and ligated into the blunt-ended p398-SKm *Eco*I site to create p398-SKmsac. This plasmid was electroporated into SPC9018 cells and the resultant kanamycin- and sucrose-resistant recombinant, SPC9018-S, was selected. Southern blot analysis was performed to verify correct insertion of the 6.1 kb fragment containing Km^R^ in the *hrpS* locus in the genetic backgrounds isolated (data not shown), showing that SPC9018-S was an *hrpS*-deficient mutant of SPC9018.

### Virulence assays.

Eggplant (*Solanum melongena* L. cv. Senryo no. 2), lettuce (*Lactuca sativa* L. cv. Success), celery (*Apium graveolens* L. cv. Topseller), sweet pepper (*Capsicum annuum* cv. Shosuke) and okra plants (*Abelmoschus esculentus* cv. Gulliver) were grown in pots containing commercial soil (Tsuchitaro, Sumitomo Forestry) in a growth room at 25 °C. Light (16 h day^−1^) was supplied at 10 000 lux throughout the experimental period. Five-week-old test plants were inoculated by leaf infiltration using a 1 ml disposable syringe with 1.0×10^8^ c.f.u. ml^−1^ bacteria in a 20 μl volume. For all assays, inoculum concentrations were determined spectrophotometrically and confirmed by dilution plating. Lettuce plants were coded and inspected for symptoms daily for 7 days after inoculation. Plants were rated on a zero-to-three disease index scale: 0, no symptoms; 1, discolouring; 2, browning; 3, collapse. Other plants were coded and inspected for symptoms daily for 7 days after inoculation. Plants were rated on a zero-to-three disease index scale: 0, no symptoms; 1, discolouring at inoculated sites; 2, necrosis at inoculated sites; 3, necrosis at the periphery of the inoculate sites. Within each trial, 12 plants of each strain were treated, yielding 60 plants per strain.

### Bacterial population *in planta*.

Areas (1 cm^2^) inoculated with *P. cichorii* strains were excised from eggplant and lettuce leaves of five plants at 0, 1, 2 and 3 days after inoculation and ground using a mortar and pestle. Samples (0.1 ml) of the original solution and 10-fold serial dilutions thereof were spread onto three plates of selective agar media of PCSM (Uematsu *et al.*, 1982) for SPC9018, media containing 50 μg kanamycin ml^−1^ for SPC9018-L and 4-57, and media containing 50 μg kanamycin ml^−1^ and 30 μg tetracycline ml^−1^ for 4-57F. Colonies were counted after 2 days of incubation at 30 °C to estimate the population.

## RESULTS

### Selection of mutants losing virulence on eggplant

**Fig. 1. f1:**
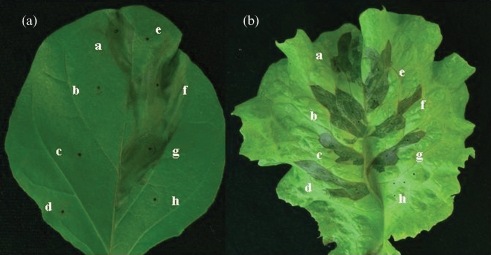
Necrotic lesions on eggplant leaves (a) and rot on lettuce leaves (b) after inoculation with *P. cichorii* strains. Five-week-old plants were inoculated by leaf infiltration with *P. cichorii* strains. a, SPC9018; b, 2-99; c, 4-57; d, SPC9018-S; e, SPC9018-L; f, 4-57F; g, 4-57FPV. Inoculates consisted of 1.0×10^8^ c.f.u. ml^−1^ bacteria and distilled water (h) in a 20 μl volume. Plants were grown in a growth room at 25 °C (10 000 lux, 16 h per day) for 3 days.

**Fig. 2. f2:**
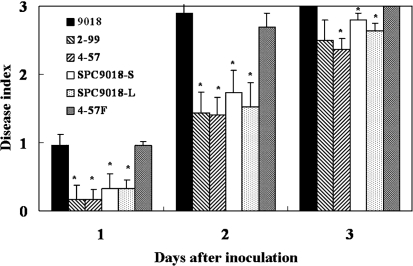
Virulence of *P. cichorii* strains on lettuce. Values represent the mean±sd of five separate experiments. Asterisks denote values significantly different from the disease index of inoculation with *P. cichorii* strain SPC9018 (*P<0.05).

SPC9018 caused necrotic lesions (Fig. 1a[Fig f1]) on eggplant and rot on lettuce (Fig. 1b[Fig f1]) within 3 days after inoculation (Fig. 2). In total, 849 and 731 transposon mutants were derived from SPC9018 by random EZ : : Tn <KAN-2> and mini-Tn*5*-insertion, respectively. We selected two mutants, 2-99 and 4-57, that were unable to cause necrotic lesions on eggplant within 7 days of inoculation (Fig. 1a[Fig f1]). However, the mutants retained their ability to cause rot on lettuce leaves, although rot symptoms caused by the mutants were delayed compared to those caused by SPC9018 (Fig. 2). Other mutants retained similar virulence to SPC9018 against both eggplant and lettuce.

### Cloning of the transposon-containing fragment from mutants

**Fig. 3. f3:**
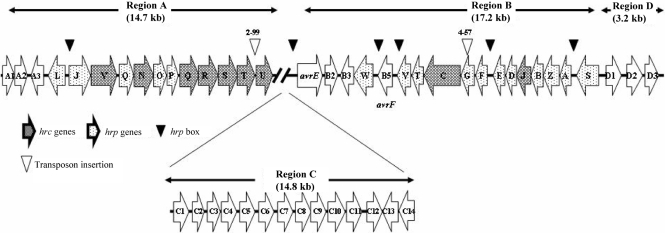
Assembly of ORFs of the DNA region that includes the *hrp* genes of *P. cichorii* strain SPC9018 genomic DNA.

In Southern analysis a single transposon insertion was observed in the DNA of 2-99 and 4-57 (data not shown). No hybridization signal was observed in the DNA of parental strain SPC9018. The *Kpn*I-digested 13.9 and 5.6 kb fragments containing the inserted region of transposons from the genomes of 2-99 and 4-57 were cloned into pUC118, and p2-99 and p4-57 were created, respectively. The nucleotide sequences of inserted fragments showed that the transposons were inserted into the *hrcT* gene in p2-99 and the *hrpG* gene in p4-57 (Fig. 3).

### Sequence analysis of the *hrp* cluster

The *Sau*3AI-digested 30.0 and 24.3 kb fragments containing *hrpG* and *hrcC* from the genome of SPC9018 were cloned into pBluescript II KS+, and pHOJO and pKAJI were created, respectively. Inserts of pHOJO and pKAJI were sequenced and ORFs were analysed based on the nucleotide sequence of *hrp* genes in 83-1. Analyses of nucleotide sequences of the 30.0 and 24.3 kb inserts showed that the DNA region including the *hrp* cluster of SPC9018 consisted of 49 ORFs. These ORFs were divided into four regions: A, B, C and D (Table 2, Fig. 3).

Fifteen ORFs were present in region A (14.7 kb). These encoded a LysR family transcriptional regulator, aldehyde dehydrogenase, phosphinothrin *N*-acetyltransferase, HrpL, HrpJ, HrcV, HrpQ, HrcN, HrpO, HrpP, HrcQ, HrcR, HrcS, HrcT and HrcU. Each ORF (Table 2, Fig. 3) was preceded by a ribosome-binding site. Region B (17.2 kb) included 17 ORFs encoding HrpS, HrpA, HrpZ, HrpB, HrcJ, HrpD, HrpE, HrpF, HrpG, HrcC, HrpT, HrpV, AvrF, HrpW, HrpW-specific chaperone, asparaginyl beta-hydroxylase and AvrE. Nucleotide sequences and alignments of these ORFs located in regions A and B showed homology to those of 83-1 and *P. viridiflava* BS-type strain RMX3.1b. Comparison to 83-1 sequences (Araki *et al.*, 2006) showed that the consensus sequence of the *hrp* box may be GGAACC-N_15-16_-CCANNCA, which was identified at 84 bp upstream of *hrpA*, 31 bp upstream of *hrpF*, 135 bp upstream of *hrpW*, 79 bp upstream of *avrF* and 61 bp upstream of *hrpJ*. Putative Rho-independent terminators were located between *hrpS* and *hrpA* and between *hrpL* and the phosphinothricin *N*-acetyltransferase gene, but no others were found.

Region C (14.8 kb) was located between region A and region B, and contained 14 ORFs (Table 2, Fig. 3). Nucleotide sequences of 12 ORFs, C1–C12, were homologous to those of CV_1407–CV_1396 of *Chromobacterium violaceum* strain ATCC 12472 (Brazilian National Genome Project Consortium, 2003). Furthermore, nucleotide sequences of C14 were homologous to those of Pput1855 of *Pseudomonas putida* strain F1. In comparison with region C of *P. cichorii* strain 83-1, one ORF, C13, existed in SPC9018 alone. The nucleotide sequence of C13 at position 94–825 showed homology to the PSPTO_3183-encoded pirin from *Pseudomonas syringae* pv. *tomato* strain DC3000 at position 31–762 (Buell *et al.*, 2003). The nucleotide sequence of C13 at 1196–1569 showed homology to the Xcc0038-encoded FND-dependent NADH azoreductor from *Xanthomonas campestris* pv. *campestris* strain ATCC 33913 at position 347–720 (da Silva *et al.*, 2002).

Three ORFs were present in region D (3.2 kb), encoding two amino acid permeases and the binding protein component of an ABC transporter, which showed homology to those of *Pseudomonas aeruginosa* strain PAO1 (Stover *et al.*, 2000) (Table 2, Fig. 3).

### Complementation of 4-57 with the *hrpF* operon

To confirm the involvement of the TTSS in *P. cichorii* virulence against eggplant, the *hrpT* mutant with the transposon insertion 4-57 was transformed with a plasmid carrying the *hrpF* operons from the SPC9018 genome. The transformant 4-57F (Figs 1a[Fig f1] and 2) showed virulence against both eggplant and lettuce plants, similar to SPC9018. Furthermore, 4-57 was transformed with phrpFoperonPV containing the *hrpF* operon from the Pv9504 genome, and the transformant 4-57FPV was created (Fig. 2). 4-57FPV showed virulence against both eggplant and lettuce plants. These results suggest that the *hrpF* operon is involved in the virulence of SPC9018 against eggplant, and that the *hrpF* operon from Pv9504 is functional in SPC9018.

### Virulence of *hrpL and hrpS* mutants

To analyse the involvement of *hrpS* and *hrpL* in the pathogenicity of SPC9018, *hrpS*- and *hrpL*-deficient mutants were created. Both the *hrpS*- and the *hrpL*-deficient mutants lost their virulence on eggplant, in a manner similar to 2-99 and 4-57 (Fig. 1a[Fig f1]). Both the *hrpS* mutant and the *hrpL* mutant retained their virulence on lettuce leaves, although rot symptoms caused by the mutants were delayed compared to those caused by SPC9018 (Figs 1b[Fig f1] and2). All *hrp* mutants lost their virulence on celery, okra and sweet pepper.

### Population of *P. cichorii* strains in eggplant and lettuce leaves

**Fig. 4. f4:**
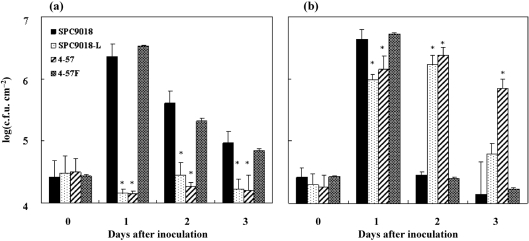
Population dynamics of *P. cichorii* strains in infiltrated eggplant leaves (a) and lettuce leaves (b). Values represent the mean±sd of five separate experiments. Asterisks denote values significantly different from the population of *P. cichorii* strain SPC9018 (*P<0.05).

Populations of 4-57 and the *hrpL*-deficient mutant SPC9018-L showed little change after inoculation into eggplant leaves, remaining at 1.4×10^4^–3.2×10^4^ c.f.u. cm^−2^ at 3 days after inoculation. On the other hand, 1 day after inoculation the parent strain reached its maximum population size of 2.3×10^6^ c.f.u. cm^−2^, and 4-57F reached its maximum of 3.4×10^6^ c.f.u. cm^−2^ (Fig. 4a[Fig f4]).

SPC9018 and 4-57F grew vigorously in lettuce leaves, and reached 4.3×10^6^ and 5.2×10^6^ c.f.u. cm^−2^ respectively, by 1 day after inoculation. 4-57 and SPC9018-L grew more slowly in lettuce leaves than the parent strain, and reached population sizes of 2.4×10^6^ and 1.7×10^6^ c.f.u. cm^−2^, respectively, by 2 days after inoculation (Fig. 4b[Fig f4]).

The parental and the mutant strains grew similarly in both PS medium and PCSM medium (data not shown).

## DISCUSSION

Lettuce leaf tissues inoculated with *P. cichorii* show browning and collapse symptoms. *P. cichorii* also causes necrotic leaf spots and induces cell death in eggplant. Our previous studies showed that there may be differences not only in the induction kinetics and level of plant responses, but also in the infection-related responses between bacterial rot in lettuce leaves and necrotic leaf spots in eggplant leaves (Kiba *et al.*, 2006a, b). The results of the present study showed that the *hrp*-deficient mutants lost their ability to grow vigorously in eggplant leaves and also their virulence towards eggplant, indicating that the virulence of *P. cichorii* towards eggplant is dependent upon the *hrp* genes. On the other hand, the *hrp*-deficient mutants retained their virulence towards lettuce. Therefore, the virulence functions of *P. cichorii* in lettuce differ from those in eggplant.

The nucleotide sequences of the *P. cichorii* *hrp* genes and their genetic structure were homologous to those of *P. viridiflava* BS group strains, suggesting a common ancestor of *hrp* clusters between *P. viridiflava* BS group strains and *P. cichorii* strains. *P. viridiflava* harbours two structurally distinct and highly diverged pathogenicity island (PAI) paralogues, T-PAI and S-PAI. These paralogues are integrated at different chromosome locations in the genome of *P. viridiflava* AT group strains, and AS group and BS group strains, respectively (Araki *et al.*, 2006). Phylogenetic analysis showed that the time of the most recent common ancestor of T-PAI and S-PAI predates the split of *P. viridiflava* from other *Pseudomonas* species. This indicates that one of the PAIs cannot have originated as a recent duplication event of the other, and a recent horizontal gene transfer cannot explain the presence/absence polymorphism of S-PAI or T-PAI. Phylogenetic analysis using nucleotide sequences of *gyrB* and *rpoD* demonstrates that within the ‘*P. syringae* complex’, *P. cichorii* strains form an independent monophyletic cluster with two strains of *P. syringae* pv. *syringae* (Yamamoto *et al.*, 2000). Our preliminary phylogenetic analysis using nucleotide sequences of *hrpL* and *hrpS*/*hrpA* also showed that 12 strains of *P. cichorii*, including SPC9018 and 83-1, form a monophyletic cluster independent of *P. viridiflava* and *P. syringae* (data not shown). It was thus thought that the time of the most recent common ancestor between S-PAI of *P. viridiflava* and the *hrp* clusters of *P. cichorii* may predate the split of *P. cichorii* from other *Pseudomonas* species. The ORFs C1–C12 and C14 in region C may have been acquired subsequently in common with *C. violaceum* and *P. putida*, respectively. The existence of ORF C13 in SPC9018 but not 83-1 suggests diversity among *P. cichorii* strains.

There are distinct differences in virulence and host-specificity between AT group strains, and AS and BS group strains of *P. viridiflava*. Araki *et al.* (2006) have suggested that these differences may be maintained by selection as alternative means of interacting with different hosts. The host range of *P. cichorii* differs from that of *P. viridiflava*, although both pathogens infect lettuce and okra. The *hrpF* operon from *P. viridiflava* BS group strain 9504 was able to complement the virulence of *hrpG* mutants on eggplants, suggesting functional conservation of *hrpF* operons between SPC9018 and Pv9504. Comparing SPC9018 and *P. viridiflava* strain RMX3.1b, the amino acid sequence identities of the TTSS-dependent effectors AvrE and HrpW were 33.6 and 61.8 %, respectively (data not shown). Furthermore, the *hrp* mutants of SPC9018 lost their ability to cause necrotic spots on not only eggplant but also celery, sweet pepper and okra. Therefore, after acquisition of the the DNA region that includes the *hrp* genes, variation of TTSS effector genes under valance selection may lead to the acquisition by *P. cichorii* of virulence against eggplant, celery, sweet pepper and okra. This would result in differences of virulence between *P. cichorii* and the *P. viridiflava* BS group. However, the roles of these candidates in bacterial virulence remain unclear.

The need for an *hrp* gene for virulence has been documented in both non-macerating plant pathogens and in macerating *Erwinia* spp. and *Pectobacterium* spp. In the soft-rot pathogens, *hrpN_Ech_* mutants are weakly pathogenic on chicory (Bauer *et al.*, 1994, 1995), whereas *hrpN_Ecc_* mutants are fully pathogenic on host plants (Cui *et al.*, 1996). Furthermore, plants infected with an *hrcC* mutant of *Pectobacterium* (*Erwinia*) *carotovora* subsp. *carotovora* show delayed symptom development (Rantakari *et al.*, 2001). Lehtimäki *et al.* (2003) have reported that high basal-level expression of *hrp*-regulated genes in *Pectobacterium* (*Erwinia*) *carotovora* subsp. *carotovora* has a negative impact on disease progress in the susceptible host plant *Arabidopsis thaliana*. The macerating enzymic bombardment launched by the necrotrophic *Erwinia* spp. and *Pectobacterium* spp. is a robust method of invasion compared with biotrophic bacterial species, which depend fully on the *hrp* gene functions for virulence. Although biotrophic pathogens also carry additional virulence determinants such as genes for toxins and extracellular polysaccharides, they are nonvirulent without the TTSS, at least under laboratory conditions. Therefore, the effect of the TTSS differs between necrotrophic and biotrophic pathogens. *P. cichorii* induces PCD in eggplant leaves, leading to development of necrotic leaf spots (Kiba *et al.*, 2006b). The *hrp*-deficient mutants lost their ability to grow vigorously in eggplant leaves and also their virulence on eggplants, suggesting that the bacteria have a biotrophic interaction with eggplants. SPC9018 grows vigorously in intercellular spaces after invasion through stomata in lettuce leaves. This leads to induction of PCD, resulting in induction of collapse and browning symptoms (Hikichi *et al.*, 1998; Kiba *et al.*, 2006a). Although the *hrp*-deficient mutants of SPC9018 retained their virulence on lettuce plants, the mutants grew more slowly, and the appearance of disease symptoms on infected lettuce leaves was delayed compared with the wild-type strain. Our data suggest that the bacteria have a more necrotrophic interaction with lettuce, and the *hrp* cluster plays a role in virulence at the early stages of infection into lettuce leaves, although the *hrp* genes are not directly implicated in induction of PCD. Therefore, after introduction into *P. cichorii* by horizontal gene transfer, the putative TTSS-dependent effector proteins may hinder or delay the plant defence response, giving the bacteria time to multiply before inducing PCD in lettuce leaves.

## Figures and Tables

**Table 1. t1:** Strains and plasmids used in this study

**Strain or plasmid**	**Relevant characteristics**	**Reference or source**
***E. coli* strains**		
DH5*α*	*recA1 endA1 gyrA96 thi-1 hsdR17supE44* Δ(*lac*)*U169*(*φ80lac*ΔM15)	Takara
***P. cichorii* strains**		
SPC9018	Wild-type	Kiba *et al.* (2006a)
2-99	SPC9018 derivative, carries a EZ : : Tn <KAN-2>, Km^r^	This study
4-57	SPC9018 derivative, carries a miniTn*5*lacZ2, Km^r^	This study
SPC9018-L	*hrpL*-deleted mutant of SPC9018, Km^r^	This study
SPC9018-S	*hrpS*-deleted mutant of SPC9018, Km^r^	This study
4-57F	Transformant of 4-57 with phrpFoperon, Km^r^, Tc^r^	This study
4-57FPV	Transformant of 4-57 with phrpFoperonPV, Km^r^, Tc^r^	This study
***P. viridiflava* strain**		
9504	BS type	This study
**Plasmids**		
pBluescript II	KS+, Ap^r^	Stratagene
pUC118	Amp^r^	Takara
pHSG398	Cm^r^	Takara
pLAFR3	pLAFR1 containing *Hae*II fragment of pUC8	Staskawicz *et al.* (1987)
placZ2	P18Sfi derivative containing mini-Tn*5*lacZ2, Km^r^	de Lorenzo *et al.* (1990)
pUCK191	pUC18 derivative containing Km^r^ from Tn*903*	Tsuge *et al.* (2001)
pUCD800	pUCD5 derivative containing *sacB*, Km^r^	Gay *et al.* (1985)
p2-99	13.9 kb *Kpn*I fragment containing the EZ : : Tn <KAN-2> in pUC118	This study
p4-57	5.6 kb *Kpn*I fragment containing the miniTn*5*lacZ2 in pUC118 pGEM-T	This study
pP2-99	703 bp *Sph*I- and *Xho*I-digested fragment of p2-99 in pUC118	This study
pP4-57	1.1 kb *Hin*dIII- and *Sma*I-digested fragment of p4-57 in pUC118	This study
pHOJO	30 kb fragment from SPC9018 genomic DNA in pBluescript II	This study
pKAJI	24.3 kb fragment from SPC9018 genomic DNA in pBluescript II	This study
pL3-4	870 bp *Pst*I- and *Sac*I-digested PCR fragment (3-4 fragment) in pHSG398	This study
phrpL	464 bp *Eco*RI- and *Sac*I-digested PCR fragment (1-2 fragment) in pL3-4	This study
p398-LKm	1.4 kb blunt-ended fragment of pUCK191 containing Km^R^ in phrpL	This study
p398-LKmsac	2.6 kb blunt-ended *Kpn*I fragment containing *sacB* from pUCD800 ligated into blunt-ended *Eco*RI site of p398-LKm	This study
p398 S	4.7 kb *Sma*I- and *Eco*RI-digested fragment of pHOJO in pHSG398	This study
p398-SKm	1.4 kb blunt-ended *Kpn*I fragment containing Km^R^ from pUCK191 ligated into *Eco*RV site of p398-S	This study
p398-SKmsac	2.6 kb blunt-ended fragment contaning *sacB* from pUCD800 ligated into blunt-ended *Eco*RI site of p398-SKm	This study
phrpFoperon	3.6 kb PCR fragment containing the *hrpF* operon from SPC9018 genomic DNA	This study
phrpFoperonPV	4.1 kb PCR fragment containing the *hrpF* operon from Pv9504 genomic DNA	This study

**Table 2. t2:** Homologues of open reading frames in A, B, C and D regions of pathogenicity islands of *P. cichorii* strain SPC9018

**ORF**		**Homologue**			
**Name**	**Start–stop**	**Bacterium**	**Gene**	**Locus tag**	**Product**
A1	42–920	*P. viridiflava* strain RMX3.1b		PAI-R2-ORF5	LysR family transcriptional regulator
A2	1078–2661		*aldH*	PAI-R2-ORF6	Aldehyde dehydrogenase
A3	Complement (2868–3449)			PAI-R2-ORF7	Phosphinothricin *N*-acetyltransferase
A4	Complement (3449–3988)		*hrpL*	PAI-R2-ORF8	HrpL
A5	4268–5386		*hrpJ*	PAI-R2-ORF9	HrpJ
A6	5383–7488		*hrcV*	PAI-R2-ORF10	HrcV
A7	7499–8482		*hrpQ*	PAI-R2-ORF11	HrpQ
A8	8563–9837		*hrcN*	PAI-R2-ORF12	HrcN
A9	9824–10 288		*hrpO*	PAI-R2-ORF13	HrpO
A10	10 285–10 845		*hrpP*	PAI-R2-ORF14	HrpP
A11	10 847–11 911		*hrcQ*	PAI-R2-ORF15	HrcQ
A12	11 980–12 561		*hrcR*	PAI-R2-ORF16	HrcR
A13	12 576–12 836		*hrcS*	PAI-R2-ORF17	HrcS
A14	12 838–13 635		*hrcT*	PAI-R2-ORF18	HrcT
A15	13 660–14 745		*hrcU*	PAI-R2-ORF19	HrcU
B1	29 511–35 000		*avrE*	PAI-R2-ORF20	AvrE
B2	35 111–35 719			PAI-R2-ORF21	Aspartyl beta-hydroxylase
B3	Complement (35 966–36 295)			PAI-R2-ORF23	HrpW-specific chaperone
B4	Complement (36 297–37 982)		*hrpW*	PAI-R2-ORF24	HrpW
B5	Complement (38 129–38 527)		*avrF*	PAI-R2-ORF25	AvrF
B6	Complement (38 629–38 979)		*hrpV*	PAI-R2-ORF26	HrpV
B7	Complement (38 972–39 163)		*hrpT*	PAI-R2-ORF27	HrpT
B8	Complement (39 291–41 309)		*hrcC*	PAI-R2-ORF28	HrcC
B9	Complement (41 302–41 715)		*hrpG*	PAI-R2-ORF29	HrpG
B10	Complement (41 702–41 950)		*hrpF*	PAI-R2-ORF30	HrpF
B11	Complement (41 994–42 572)		*hrpE*	PAI-R2-ORF31	HrpE
B12	Complement (42 538–43 161)		*hrpD*	PAI-R2-ORF32	HrpD
B13	Complement (43 158–43 949)		*hrcJ*	PAI-R2-ORF33	HrcJ
B14	Complement (43 960–44 328)		*hrpB*	PAI-R2-ORF34	HrpB
B15	Complement (44 364–45 386)		*hrpZ*	PAI-R2-ORF35	HrpZ
B16	Complement (45 411–45 617)		*hrpA*	PAI-R2-ORF36	HrpA
B17	Complement (45 735–46 673)			PAI-R2-ORF37	HrpS/HrpR-like protein
C1	15 379–16 395	*C. violaceum* strain ATCC 12472		CV_1407	Dehydrogenase
C2	16 429–17 544		*degt*	CV_1406	Aspartate aminotransferase
C3	17 545–18 057			CV_1405	Unknown
C4	18 054–19 142			CV_1404	Aminotransferase
C5	19 123–20 493			CV_1403	Unknown
C6	20 490–21 188			CV_1402	Phosphoglycolate phosphatase
C7	21 191–22 303			CV_1401	Dihydrorhizobitoxine desaturase
C8	22 393–23 208			CV_1400	5′-Methylthioadenosine phosphorylase
C9	23 205–23 801			CV_1399	Unknown
C10	23 810–24 724			CV_1398	Unknown
C11	24 721–25 908			CV_1397	Beta-phosphoglucomutase
C12	25 915–26 571			CV_1396	ABC transporter
C13	Complement (26 678–28 246)	*P. syringae* pv. *tomato* strain DC3000/*X. campestris* pv. *campestris* strain ATCC 33913		PSPTO_3183/Xcc0038	Pirin/FND-dependent NADH azoreductor
C14	Complement (28 375–29 121)	*P. putida* strain F1		Pput_1855	Unknown
D1	47 215–48 021	*P. aeruginosa* strain PAO1		PA2204	Binding protein component of ABC
D2	48 090–48 830			PA2203	Amino acid permease
D3	48 833–49 519			PA2202	Amino acid permease

## Abbreviations

- PCD, programmed cell death
- TTSS, type III secretion system
